# Priming seeds for the future: Plant immune memory and application in crop protection

**DOI:** 10.3389/fpls.2022.961840

**Published:** 2022-07-29

**Authors:** Zige Yang, Pengfei Zhi, Cheng Chang

**Affiliations:** College of Life Sciences, Qingdao University, Qingdao, Shandong, China

**Keywords:** seed priming, immune memory, epigenetics, disease resistance, crop protection

## Abstract

Plants have evolved adaptive strategies to cope with pathogen infections that seriously threaten plant viability and crop productivity. Upon the perception of invading pathogens, the plant immune system is primed, establishing an immune memory that allows primed plants to respond more efficiently to the upcoming pathogen attacks. Physiological, transcriptional, metabolic, and epigenetic changes are induced during defense priming, which is essential to the establishment and maintenance of plant immune memory. As an environmental-friendly technique in crop protection, seed priming could effectively induce plant immune memory. In this review, we highlighted the recent advances in the establishment and maintenance mechanisms of plant defense priming and the immune memory associated, and discussed strategies and challenges in exploiting seed priming on crops to enhance disease resistance.

## Introduction

Plants employ a plethora of mechanisms to defend against invading pathogens, including virus, bacteria, fungi, oomycetes, and pests (Zhou and Zhang, 2020). Thorns, spikes, cuticles, cell walls, and antimicrobial secondary metabolites constitute the plant preformed defense to deter pathogens. As an inducible defense mechanism, pattern-triggered immunity (PTI) is initiated by cell surface-localized pattern-recognition receptors (PRRs) upon the perception of pathogen patterns. In addition, plants utilize intracellular nucleotide-binding domain leucine-rich repeat-containing receptors (NLRs) to detect pathogen effector proteins and activate effector-triggered immunity (ETI; Yu et al., 2017; Saur et al., 2021). PTI and ETI are initiated by different activation mechanisms and usually have distinct dynamics and amplitude. Recent studies revealed that PTI and ETI converge into some common downstream signaling pathways and potentiate each other in the unified plant immunity (Ngou et al., 2021; Yuan et al., 2021a,b).

During long-term coevolution with pathogens, plants have acquired adaptive strategies to cope with recurrent pathogen infections. Perception of initial pathogens by plants could induce a primed state marked by the enhanced activation of defense responses upon the subsequent pathogen challenges (Reimer-Michalski and Conrath, 2016; Mauch-Mani et al., 2017). This defense priming is typically associated with induced resistance (IR) such as systemic acquired resistance (SAR), induced systemic resistance (ISR), and mycorrhiza-induced resistance (MIR; Reimer-Michalski and Conrath, 2016; Mauch-Mani et al., 2017). Defense priming requires immune memory to store the changes or information acquired from the initial pathogen perception, and retrieves this information upon a later pathogen challenge (Ramirez-Prado et al., 2018). As an environmental-friendly, pre-sowing enhancement technique, seed priming could effectively induce plant immune memory and have a great potential in sustainable crop protection (Jogaiah et al., 2020; Joshi et al., 2021; Martínez-Aguilar et al., 2021; Pal et al., 2021; Yadav et al., 2021; Kappel et al., 2022). Herein, we summarized recent development on the establishment and maintenance mechanisms of plant defense priming and the immune memory associated. Strategies, limitations, and future directions in exploiting seed priming for crop protection are discussed.

## Plant defense priming and immune memory

Primed state of plant immune system could be induced by various biological, physical, and chemical stimuli. Typically, pathogens and their derived molecules such as patterns and effectors could act as warning signals to trigger plant defense priming (Abdul Malik et al., 2020). Furthermore, beneficial interactions with root-colonizing microorganisms could lead to the establishment of primed state (Yu et al., 2022). Moreover, herbivore-associated signals such as physical contacts, oral secretions, and oviposition fluids could function as priming stimuli (Mauch-Mani et al., 2017). Interestingly, certain abiotic stresses such as extreme temperatures and mechanical wounding could prime the plant immune system (cross-priming; Liu et al., 2022). Defense-related phytohormones jasmonic acid (JA), salicylic acid (SA), and their derivatives could induce plant defense priming when applied exogenously (Mauch-Mani et al., 2017). Synthetic functional SA analogs N-cyanomethyl-2-chloro isonicotinic acid (NCI), benzothiadiazole (BTH)/acibenzolar-S-methyl (ASM), and isotianil are potent priming inducers. In addition, a plethora of plant metabolites and related synthetic chemicals such as sulforaphane (SFN), β-amino acids (R)-beta-homoserine (RBH), glycerol, and enzyme ascorbate oxidase (AO) were recently identified as defense priming agents (Buswell et al., 2018; Zhou and Wang, 2018; Li et al., 2020; Singh et al., 2021). Due to their unique physicochemical properties, nanomaterials such as nanoparticles and nanoemulsions are increasingly employed in plant defense priming (Do Espirito Santo Pereira et al., 2021). Notably, functional SA analog BTH/ASM, non-protein amino acid β-aminobutyric acid (BABA), and chitin polymeric derivative chitosan have been successfully developed into commercial priming agents (Yassin et al., 2021).

Upon perception of initial priming stimuli, the plant would enter into the priming phase and undergo physiological, transcriptional, metabolic, and epigenetic changes (Mauch-Mani et al., 2017). Although most of these changes are transient and disappear quickly after the initial stimuli were removed, some alterations could be retained to form plant somatic immune memory (Lämke and Bäurle, 2017). In a few cases, these changes occur in plant reproductive tissues including gametes to form intergenerational or transgenerational immune memory. Generally, plant intergenerational immune memory is unstable during meiosis and affects only one stress-free generation. In contrast, plant transgenerational immune memory is meiotically stable and could be detected in two or more stress-free generations (Ramírez-Carrasco et al., 2017).

## Physiological, transcriptional, and metabolic changes during plant defense priming

After perception of invading pathogens, plants induce defense responses such as elevation in cytoplasmic calcium concentration ([Ca^2+^]_cyt_), ROS burst, and callose deposition (Balmer et al., 2015; Cao et al., 2017; Hake and Romeis, 2019). Defense-related calcium changes were reported in various plant cells or tissues in response to the treatment with synthetic PAMPs oligopeptide flg22, pep13, liposaccharides, and chitin (Balmer et al., 2015). Interestingly, noctuid moth (*Spodoptera littoralis*) feeding could induce a systemic [Ca^2+^]_cyt_ elevation in *Arabidopsis*, but this calcium response in *Arabidopsis* systemic tissues was not observed upon exposure to the synthetic PAMP flg22 (Cao et al., 2017). Pretreatment with polypeptide extract from dry mycelium of *Penicillium chrysogenum* (PDMP) could induce disease resistance against tobacco mosaic virus (TMV) in tobacco plants (Li et al., 2021b). Recent RNA sequencing (RNA-seq) and fluorescence microscopy demonstrated that pretreatment with PDMP inhibited TMV movement by increasing callose deposition around plasmodesmata (Li et al., 2021b). However, PDMP-induced callose deposition was not observed in the ABA biosynthesis mutant, which could be rescued by exogenous ABA treatment (Li et al., 2021b). These results suggested that PDMP-pretreatment induced ABA biosynthesis-dependent callose priming to protect tobacco plants from TMV infection (Li et al., 2021b).

Massive transcriptional reprogramming has been reported to take place in response to pathogen infections and priming agent treatments in model and crop plants. Although enhanced resistance against *P. syringae* pv. *phaseolicola* infection was induced by non-protein amino acid BABA and SA analog INA in common bean (*P. vulgaris*), but BABA and INA primed different defense-related genes, suggesting that distinct transcriptomic reprogramming takes place in response to different priming stimuli (Martínez-Aguilar et al., 2016). Consistent with this, a transcriptomic analysis showed that 33 genes were specifically induced by the priming agent sulfated laminarin (PS3) but not by laminarin (Lam) in grapevine (*Vitis vinifera*; Gauthier et al., 2014). Transcriptomic reprogramming induced by priming stimuli ultimately results in massive proteomic changes in primed plants. Indeed, accumulation of MPK3/6, PR proteins, pattern recognition receptor FLS2, and coreceptor BAK1 was primed by BABA and BTH treatment in *Arabidopsis thaliana*, *Lactuca sativa*, and *Solanum tuberosum* (Beckers et al., 2009; Tateda et al., 2014; Baccelli and Mauch-Mani, 2016). Some of these transcriptomic and proteomic changes would confer primed plant enhanced responsiveness to the subsequent pathogen infections.

To prepare for the incoming pathogens, primed plants usually undergo metabolic changes in the biosynthesis of primary and secondary metabolites (Frost et al., 2008; War et al., 2011; Brosset and Blande, 2022). It was recently demonstrated that BABA treatment induced resistance to *Botrytis cinerea* and affected the contents of soluble sugar and phenylpropanoid metabolites in grape berries (Li et al., 2021a). RNA-seq and comparative transcriptomic analysis revealed that treatment of grapes with 100 mM BABA relatively upregulated genes associated with phenylpropanoid biosynthesis compared with grapes subjected to 10 mM BABA treatment. These results suggested that the BABA-primed defense determines alterations in sucrose and phenylpropanoid metabolism in postharvest grapes (Li et al., 2021a). Interestingly, the grape MYB-type transcription factor VvMYB44 directly activates the expression of sucrose and phenylpropanoid metabolism-related genes, and might participate in BABA-induced priming (Li et al., 2021a).

## Epigenetic mechanisms of plant defense priming

In plants, methylation of cytosine to 5-methylcytosine (5-mC) mainly occurs in the sequence context of CG, CHG, and CHH (H is A, C, or T; Zhang et al., 2006; Elhamamsy, 2016; Kong et al., 2020; Zhi and Chang, 2021). Plant DNA cytosine methylation profile is initially established *via* the RNA-dependent DNA methylation (RdDM) pathway involving the DNA methyltransferase DOMAINS REARRANGED METHYLTRANFERASE 2 (DRM2), and maintained by DNA methyltransferases METHYLTRANSFERASE 1 (MET1), CHROMOMETHYLASE 2 (CMT2) and CMT3 during mitosis and meiosis (Yaari et al., 2019; Erdmann and Picard, 2020). As a reversible epigenetic mark, 5-mC could be directly removed by DNA glycosylases such as REPRESSOR OF SILENCING 1 (ROS1), DEMETER (DME), DEMETER-LIKE 2 (DML2), and DML3 in *Arabidopsis* (Zhu, 2009; Tang et al., 2016). DNA methylation occurs in various genomic regions including gene promoters and transposable elements (Chan et al., 2005; Law and Jacobsen, 2010). Generally, gene promoter hypermethylation is associated with gene repression, whereas transposable element hypermethylation contributes to the TEs silencing and genome stability maintenance (Elhamamsy, 2016; Zhi and Chang, 2021). Genome-wide DNA hypomethylation induced by invading pathogens and/or priming agents has been widely observed in a wide range of plant species, which has been extensively discussed in prior reviews (Atighi et al., 2020; Annacondia et al., 2021; Zhi and Chang, 2021; Huang and Jin, 2022).

Although DNA cytosine methylation usually affects the expression of nearby defense genes *in cis*, *in trans*-regulation by DNA methylation might be more important to plant defense priming (van Hulten et al., 2006). Treatment of priming agent BABA leads to a genome-wide DNA cytosine hypomethylation in tomatoes (Catoni et al., 2022). DNA methylome and transcriptome analysis revealed that about 80% of primed tomato genes did not contain any differentially methylated regions (DMRs), suggesting that DNA cytosine methylation regulates the majority of defense-related transcription *in-trans* (Catoni et al., 2022). *Pst*DC3000-triggered SAR is transmitted to at least two stress-free generations, and this transgenerational SAR was potentiated in the DNA hypomethylation mutant *dmr1dmr2ctm3* (*ddc*; Luna et al., 2012). This study supports the involvement of DNA cytosine methylation in the generational transmission of plant immune memory. Consistent with this, DNA cytosine methylation at the promoter region of the *R3a* resistance gene is associated with the potato intergenerational resistance against late blight disease (Meller et al., 2018). In *Arabidopsis*, mitochondrial stress (MS) triggered by exogenous applications of antimycin A (AA) could induce plant resistance (MS-IR) against the biotrophic oomycete pathogen *Hyaloperonospora arabidopsidis* (*Hpa*; López Sánchez et al., 2021). It was demonstrated that the MS-IR could be transmitted to one stress-free generation (López Sánchez et al., 2021). Notably, this intergenerational MS-IR is compromised in the DNA hypomethylation mutant *nrpe1* and DNA hypermethylation mutant *ros1*, implicating that DNA cytosine (de)methylation machinery gets involved in the generational transmission of MS-IR (López Sánchez et al., 2021).

N-terminal histone tails stretching out of the nucleosome core could be subject to various modifications such as acetylation and methylation (Imhof and Wolffe, 1998; Tessarz and Kouzarides, 2014; Liu and Chang, 2021; Peng et al., 2021). Histone acetylation catalyzed by histone acetyltransferase (HAT) usually facilitates gene transcription, whereas histone deacetylation mediated by histone deacetylase (HDAC) could repress gene expression. In contrast, histone methylation co-regulated by histone methyltransferase and histone demethylase contributes to both gene repression and activation. Generally, H3K4me3 and H3K36me3 act as active chromatin marks, whereas H3K9me3 and H3K27me3 are linked to repressive chromatin states (Black et al., 2012). Chromatin immunoprecipitation (ChIP) analysis revealed enrichment of permissive chromatin marks H3K4me3 and H3K36me3 at defense-associated genes was induced by BABA and INA treatments in the common bean (Martínez-Aguilar et al., 2016, 2021). Notably, BABA application could induce the bistable deposition of permissive mark H3K4me2 and repressive mark H3K27me3 on defense-related genes *Non-expressor of PR genes* (*NPR1*) and *Suppressor of NPR1* (*SNI1*) in potato (Meller et al., 2018). This switchable chromatin state was proposed to be associated with the enhanced responsiveness of defense genes in primed plants (Meller et al., 2018).

Functional characterization of histone-modifying enzymes sheds novel light on the epigenetic regulation of plant defense priming and immune memory. AtLDL1 and AtLDL2 were identified as two *Arabidopsis* homologs of human lysine-specific demethylase1-like1 (LDL1; Noh et al., 2021). The *ldl1 ldl2* double mutant displayed increased H3K4me1 accumulation at the promoter regions of defense-related genes, potentiated defense-related transcription, and enhanced disease resistance against the secondary *Pseudomonas* infection (Noh et al., 2021). This evidence supports that LDL1 and lLDL2 negatively regulate the defense priming *via* the epigenetic suppression of defense-related genes (Noh et al., 2021). The contribution of histone modification to the generational transmission of plant immune memory has been supported by current evidence. BABA treatment could enhance the potato resistance against the oomycete pathogen *P. infestans*, and this pronounced disease resistance could be transmitted to at least one stress-free generation (Meller et al., 2018). Notably, the enhanced deposition of permissive epigenetic mark H3K4me2 was observed at SA-responsive genes such as *StPR1* and *StPR2* in both BABA-primed (F0) parent plant and its progeny (F1) in the absence of *P. infestans* challenge (Meller et al., 2018). This study revealed that the epigenetic mark H3K4me2 might contribute to the generational transmission of immune memory in potatoes (Meller et al., 2018).

In response to developmental and environmental cues, chromatin structure is dynamically and tightly regulated by various modulators such as histone chaperones and chromatin remodelers (Zhou et al., 2015; Song et al., 2021). As a major histone chaperone, CHROMATIN ASSEMBLY FACTOR 1 (CAF-1) could associate with the replisome and gets involved in the *de novo* assembly of histone H3 and H4 into nucleosomes (Han et al., 2015; Mozgová et al., 2015; Muñoz-Viana et al., 2017). Nucleosome occupancy micrococcal nuclease (MNase) assays revealed low nucleosome enrichment at common bean (*P. vulgaris*) *PATHOGENESIS RELATED GENE-1* gene (*PvPR1*) was induced by either INA treatment or *Pseudomonas syringae* pv. *phaseolicola* NPS3121 (*Psp*NPS3121) infection (Martínez-Aguilar et al., 2021). This study suggested that chromatin structure at defense-related genes was changed by pathogen infections and/or priming agent treatments. Consistent with this, BABA treatment and SA application both lead to reduced nucleosome occupancy at defense-related genes *PR1*, *PR5*, *WRKY6*, and *WRKY53* in *Arabidopsis* (Mozgová et al., 2015). Notably, chromatin features such as low nucleosome occupancy at defense-related genes in CAF-1 mutants *fasciata2* (*fas2*) resemble BABA-primed or SA-treated wild-type plants, suggesting that histone chaperone CAF-1 suppresses chromatin structure changes essential for plant defense priming (Mozgová et al., 2015). In addition to histone chaperons, chromatin remodelers regulate chromatin structure changes in plant defense response and priming. Chromatin remodeling factor DDM1 is a SWI2/SNF2-like protein (Brzeski and Jerzmanowski, 2003). Loss of DDM1 functions resulted in decreased DNA cytosine methylation in the *Arabidopsis* NB-LRR-encoding genes (Li et al., 2010; Kong et al., 2018). Another *Arabidopsis* chromatin remodeling factor MOM1 was demonstrated to regulate the expression of immune receptor genes by targeting distal pericentromeric transposable elements (Cambiagno et al., 2018). Interestingly, treatment with priming compound BIT (1,2-benzisothiazol-3(2 h)-one,1, 1-dioxide) in rice could inhibit the expression of the rice chromatin remodeler gene *BRHIS1*, and attenuate the suppression of BRHIS1 on defense-related transcription (Li et al., 2015). This study suggested a potential role of chromatin remodeler BRHIS1 in repressing chromatin remodeling required for defense priming in rice (Li et al., 2015).

## Strategies and challenges in exploiting seed priming to improve crop disease resistance

Seed priming is a feasible, pre-sowing enhancement technique and has been widely employed in the commercial production of crop seeds (Paparella et al., 2015). As extensively discussed in prior reviews, seed priming initiates multiple pre-germinative metabolisms, including enzyme activation, energy production, metabolites biosynthesis, and DNA repair (Hussain et al., 2016). Seed priming could secure the enhanced and uniformed seed germination and seedling establishment under field conditions, and greatly contributes to the improvement of crop growth and production (Marthandan et al., 2020; Johnson and Puthur, 2021). Increasing evidence revealed that seed priming could induce plant immune memory that is either stably maintained throughout developmental stages or transmitted over generations (Jogaiah et al., 2020; Joshi et al., 2021; Martínez-Aguilar et al., 2021; Pal et al., 2021; Yadav et al., 2021; Kappel et al., 2022). As summarized in Table 1, different types of seed priming approaches such as biological priming, chemical priming, and nanomaterials priming have been successfully established to protect crop plants against pathogen infections.

**Table 1:** Summary of seed priming approaches for crop disease resistance improvement.

Priming approach category	Priming stimuli	Crop species	Priming impact and pathways affected	Type of immune memory	Crop disease resistance retest treatment	References
Biological priming	*Trichoderma harzianum* TriH_JSB27	*Solanum lycopersicum*	*T. harzianum* TriH_JSB27 -primed tomato plants exhibited induction of defense-related *SlPAL* genes.	Somatic immune memory	Primed tomato plants exhibited enhanced disease resistance against *Ralstonia solanacearum*.	Jogaiah et al., 2013
	*Pseudomonas fluorescens*	*Pennisetum glaucum*	*P. fluorescens* -primed pearl millet plants exhibited significant changes in protein abundance.	Somatic immune memory	Primed pearl millet plants displayed increased disease resistance against downy mildew.	Anup et al., 2015
	*T. longibrachiatum*	*Allium cepa*	*T. longibrachiatum*-primed onion plants exhibited accumulation of stress-responsive metabolites.	Somatic immune memory	Primed onion plants exhibited enhanced disease resistance against *Fusarium oxysporum* f. sp. c*epa* (FOC) infection.	Abdelrahman et al., 2016
	*Bacillus amyloliquefaciens*, *P. fluorescens*	*Withania somnifera*	Priming of Ashwagandha with two bacteria combinations induced plant physiological and transcriptional changes.	Somatic immune memory	Primed Ashwagandha plants exhibited increased disease resistance against *Alternaria alternata*.	Mishra et al., 2018
	*T. harzianum*, *T. asperellum*, *Paenibacillus dendritiformis*	*Capsicum annuum*	Priming of chilli with *T. harzianum*, *T. asperellum*, and *P. dendritiformis* induced plant physiological, transcriptional, and metabolic changes.	Somatic immune memory	Primed chilli plants exhibited increased disease resistance against anthracnose disease.	Yadav et al., 2021
	*T. atroviride*	*Beta vulgaris*	*T. atroviride*-primed sugar beet plants exhibited upregulation of *BvPR3* gene.	Somatic immune memory	Priming of sugar beet plants with *T. atroviride* decreases the severity of CLS disease.	Kappel et al., 2022
	Heat-stable metabolites of *B. gaemokensis* strain PB69	*Cucumis sativus*, *C. annuum*	Priming of cucumber and pepper with heat-stable bacterial metabolites induced expression of defense-related genes.	Somatic immune memory	Primed cucumber and pepper plants exhibited increased resistance against *Pseudomonas syringae* pv. lachrymans.	Song et al., 2017
	Total crude protein (TCP) extract of *Trichoderma* spp.	*P. glaucum*	Priming of pearl millet with TCP from *Trichoderma* spp. enhanced levels of peroxidase and lipoxygenase	Somatic immune memory	Primed pearl millet plants displayed enhanced disease resistance against downy mildew.	Nandini et al., 2017
	Lipopolysaccharide (LPS) elicitors isolated from *P. fluorescens*	*P. glaucum*	Priming of pearl millet with LPS induced ROS burst, callose deposition, and induction of PR genes.	Somatic immune memory	Primed pearl millet plants exhibited increased disease resistance against downy mildew disease.	Lavanya et al., 2018
	Salicylic acid	*Solanum melongena*, *S. lycopersicum*	Priming of eggplant plants with SA induced expression of *MPK1*, *GPX,* and *PRs*, whereas SA seed-primed tomato plants exhibited induction of *APx*, *CAT* and *GR*.	Somatic immune memory	Primed eggplant and tomato plants exhibited increased disease resistance against Verticillium wilt, and bacterial spot disease, respectively.	Mahesh et al., 2017; Srinivasa et al., 2022
	Jasmonic acid	*S. lycopersicum*	JA-primed tomato plants exhibited enhanced expression of the JA-dependent defense gene *PinII*.	Somatic immune memory	Primed tomato plants exhibited resistance to herbivory by spider mites, caterpillars aphids, and infection of *B. cinerea*.	Worrall et al., 2012
	Methyl jasmonate	*S. lycopersicum*	MeJA seed-primed tomato plants exhibited an increase in the levels SA, kaempferol, and quercetin, upregulation of *PAL5*, *BSMT*, *CHS*, *FLS*, and downregulation of *ICS* gene.	Somatic immune memory	Primed tomato plants exhibited enhanced disease resistance to the hemi-biotroph *Fusarium oxysporum*.	Król et al., 2015
	β-aminobutyric acid (BABA)	*S. lycopersicum*, *P. glaucum*	BABA-primed pearl millet plants showed significant changes in protein abundance including the over-representation of proteins related to glucose metabolism	Somatic immune memory	Primed tomato and pearl millet plants exhibited increased disease resistance against powdery mildew and downy mildew, respectively.	Worrall et al., 2012; Anup et al., 2015
Chemical priming	Chitosan	*P. glaucum*, *C. sativus*, *B. vulgaris*	Priming of pearl millet seeds with chitosan increased levels of chitosanase, whereas chitosan-primed cucumber plants showed enhanced deposition of lignin, callose, and H_2_O_2_. Chitosan seed-primed sugar beet plants exhibited upregulation of *PR3, PAL,* and *GST* genes.	Somatic immune memory	Primed pearl millet plants exhibited increased disease resistance downy mildew, whereas primed cucumber and sugar beet plants exhibited enhanced disease resistance against powdery mildew and CLS disease, respectively.	Manjunatha et al., 2008; Jogaiah et al., 2020; Kappel et al., 2022
	2,6-dichloroisonicotinic acid (INA)	*Phaseolus vulgaris*	INA-primed common bean plants and its stress-free offsprings exhibited enrichment of H3K4me3 and H3K36me3, as well as low nucleosome occupancy at *PvPR1* gene.	Transgenerational immune memory	Primed common bean plants and its stress-free offsprings exhibited reduced susceptibility to *P. syringae* pv. *phaseolicola* pathogen.	Martínez-Aguilar et al., 2021
	Cholic acid-glycine conjugates (CAGCs)	*Oryza sativa*	Seed priming of rice plants with CAGCs induced expression of defense-related genes.	Somatic immune memory	Primed rice plants exhibited enhanced resistance against leaf blight disease.	Pal et al., 2021
Nanomaterial priming	Mycogenic selenium nanoparticles (SeNPs)	*S. lycopersicum*	SeNPs-primed tomato plants exhibited accumulations of lignin, callose, and elevated levels of LOX, PAL, GLU, and SOD.	Somatic immune memory	Primed tomato plants displayed enhanced resistance against the late blight.	Joshi et al., 2021
	Nanoemulsions formulated from membrane lipids of *Trichoderma brevicompactum* (UP-91)	*P. glaucum*	Priming of pearl millet with nanoemulsions induced deposition of lignin, enhanced expression of *LOX*, *AOC,* and *α-DOX* genes, and potentiated production of JA and MeJA.	Somatic immune memory	Primed pearl millet plants displayed enhanced resistance against the downy mildew disease.	Nandini et al., 2019

**Figure 1:**
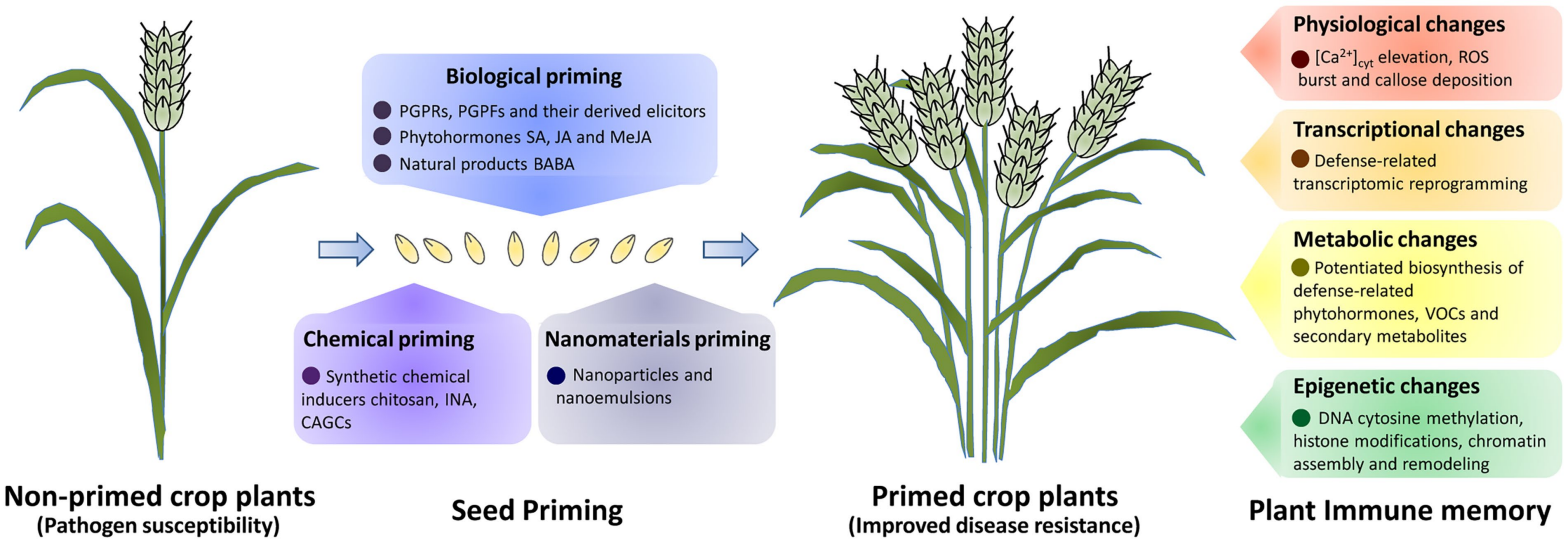
A schematic of seed priming and plant immune memory for crop disease resistance improvement. Priming of crop seeds with beneficial microbes and their derived elicitors, phytohormones and natural products, biological primimg could lead to the establishment of immune memory and induced crop disease resistance. Seed primimg with synthetic chemical inducers (chemical primimg), also could improve crop disease resistance. Physiological, transcriptional, metabolic and epigenetic changes are induced in defence priming to establish the immune memory in primed crop plants.

Beneficial microbes such as plant-growth-promoting fungi (PGPFs) *Trichoderma* spp., plant-growth-promoting rhizobacteria (PGPRs) *Pseudomonas* spp., *Paenibacillus* spp., and *Bacillus* spp. have been employed in seed primings on crops (summarized in Table 1 and Figure 1). Seed priming of chilli with PGPFs *T. harzianum*, *T. asperellum*, and PGFR *Paenibacillus dendritiformis* triggers physiological, transcriptional, and metabolic changes such as ROS burst and induction of defense-related enzymes and phenolic compounds, as well as increased disease resistance against anthracnose disease (Mitra et al., 2021; Yadav et al., 2021). Sugar beet primed with the PGPF *T. atroviride* exhibited upregulation of defense gene *BvPR3* and induced systemic resistance against Cercospora leaf spot (CLS) disease (Kappel et al., 2022). In addition, seed priming of crop plants with elicitors derived from beneficial microbes also could trigger immune memory, as well as induced resistance, throughout their developmental stages. Seed priming of pearl millet with total crude protein (TCP) extracted from *Trichoderma* spp. enhanced levels of peroxidase and lipoxygenase, and induced pearl millet disease resistance against downy mildew (Nandini et al., 2017). Pearl millet primed with LPS isolated from *Pseudomonas fluorescens* exhibited physiological and transcriptional changes such as ROS burst, callose deposition, and upregulation of *PR* genes, as well as induced disease resistance against downy mildew disease (Lavanya et al., 2018).

Phytohormones SA and JA and plant natural product BABA could effectively induce crop disease resistance when applied exogenously in seed priming (summarized in Table 1 and Figure 1). For instance, eggplant primed with SA exhibited upregulation of defense-related genes *MPK1*, *GPX*, and *PR*s, and showed increased disease resistance against *Verticillium* wilt (Mahesh et al., 2017). Priming of tomato with SA induced expression of *APx*, *CAT*, and *GR*, and enhanced bacterial spot disease resistance (Srinivasa et al., 2022). Notably, MeJA-primed tomato plants exhibited increased levels SA, kaempferol, and quercetin, upregulation of *PAL5*, *BSMT*, *CHS*, and *FLS*, as well as enhanced tomato disease resistance to the hemi-biotroph *Fusarium oxysporum* (Król et al., 2015). In addition, pearl millet primed with BABA exhibited significant changes in protein abundance and enhanced disease resistance against downy mildew (Anup et al., 2015). These studies paved a path for the exploitation of phytohormones and natural products in seed priming for crop protection.

Synthetic chemical inducers chitosan, INA, and cholic acid-glycine conjugates (CAGCs) have been successfully applied in the seed priming of crop plants for disease resistance improvement (see Table 1 and Figure 1). Priming of cucumber with chitosan induced deposition of lignin and callose, enhanced the accumulation of defense-responsive enzymes, and increased disease resistance against powdery mildew (Jogaiah et al., 2020). Chitosan-primed sugar beet plants exhibited upregulation of *PR3*, *PAL*, and *GST* genes, as well as enhanced resistance against CLS disease (Kappel et al., 2022). Rice primed with CAGCs induced expression of defense genes *EDS1*, *ICS1*, *NPR1*, *MKK4*, and *PR1* genes, and enhanced resistance against rice bacterial leaf blight disease (Pal et al., 2021). Notably, INA-primed common bean plants and their stress-free offsprings exhibited epigenetic changes such as enrichment of H3K4me3 and H3K36me3, as well as low nucleosome occupancy at *PvPR1* gene (Martínez-Aguilar et al., 2021). This study demonstrated that seed priming with INA induced the establishment of transgenerational immune memory in common bean (Martínez-Aguilar et al., 2021). Consistent with this, INA-primed common bean plants and their stress-free offsprings exhibited reduced susceptibility to the bacterial pathogen *P. syringae* pv. *phaseolicola* (Martínez-Aguilar et al., 2021). Recently, advanced chemical inducers-synthesis strategies such as computer-aided inducer design have been developed, which would certainly contribute to the advance of seed priming and its application in crop protection (Zhou and Wang, 2018).

With the advance in nanotechnology, several nanomaterials have been developed for crop protection (Do Espirito Santo Pereira et al., 2021). As summarized in Table 1 and Figure 1, nanomaterials could effectively trigger crop immune memory and induced disease resistance when applied exogenously in defense priming (Quiterio-Gutiérrez et al., 2019; Shelar et al., 2021). Seed priming of tomato with mycogenic selenium nanoparticles (SeNPs) induced accumulation of lignin and hydrogen peroxide, as well as elevated expression levels of *LOX*, *PAL*, *GLU*, and *SOD* genes (Table 1; Joshi et al., 2021). These SeNP-primed tomato plants displayed enhanced resistance against the late blight caused by *Phytophthora infestans* throughout their developmental stages, indicating that nanoparticles could be applied in the seed priming for crop protection (Joshi et al., 2021). Priming of pearl millet with nanoemulsions formulated from membrane lipids of *Trichoderma brevicompactum* (UP-91) effectively induced deposition of lignin, ROS, and callose, and increased pearl millet resistance against downy mildew disease (Table 1; Nandini et al., 2019). This study suggested that combined nanotechnology with biological priming might represent a promising seed priming method for crop protection (Table 1; Nandini et al., 2019).

To secure crop production under pathogen threats, natural and induced genetic variations have been employed for crop improvement *via* conventional or genomic breeding (Rodriguez-Moreno et al., 2017). Genetic engineering, genomic editing, and targeting induced local lesions in genomes (TILLING) of resistance or susceptibility genes represent promising approaches in crop breeding (Bruce, 2012; Acevedo-Garcia et al., 2017; Gao, 2021; Koseoglou et al., 2022). At the same time, integrated management systems based on host-pathogen-environment interaction have been established to control some pathogens and pests (Jindo et al., 2021). Compared with these current approaches, seed priming is cost and time effective, and applicable to a wide range of crop species, including those recalcitrant crops with low rates of transformation and regeneration. Furthermore, seed priming could enhance crop resistance to multiple types of pathogens. For example, BABA enhances disease resistance against powdery mildew and downy mildew in several crop species (Worrall et al., 2012; Anup et al., 2015). As discussed in the epigenetic section in detail, defense priming induced epigenetic changes such as alteration in DNA methylation, which could lead to mobilization of transposable elements and formation of heritable genetic variations (Luna et al., 2012; Meller et al., 2018; López Sánchez et al., 2021; Catoni et al., 2022). These genetic variations could be employed for breeding purposes, which might provide a direction to integrate priming strategy into breeding programs in future research.

Although seed priming has great potential for use in crop protection, caution must be exercised in the application of priming materials. The safety of priming microbes, chemicals, and nanomaterials, as well as their impact on ecosystems and fates in environments, needs to be extensively evaluated before large-scale application. Some priming chemicals such as BABA, chitosan, and BTH are commercially available, but industrial production of other priming materials such as PGPFs, RGRRs, and nanomaterials need to be established or optimized to meet the demand in agronomical practices. Since pre-treatment with some priming materials like BABA and BTH usually induces plant defense response and leads to growth penalty, it is crucial to establish proper application conditions for each priming agent (Buswell et al., 2018). In addition, priming concentration and duration also need to be optimized for each crop variety.

## Concluding remarks and perspectives

In this review, we summarized molecular bases of plant defense priming and immune memory associated, and discussed recent advances and future directions in exploiting seed priming for crop protection. As shown in Figure 1, seed priming of crop plants with beneficial microbes, phytohormones, and natural products (biological priming), synthetic chemical inducers (chemical priming), nanoemulsions, and nanoparticles (nanomaterials priming) could effectively improve crop resistance against pathogen infections. Physiological, transcriptional, metabolic, and epigenetic changes are induced by defense priming to constitute the immune memory that is either stably maintained in developmental stages or transmitted over generations in primed crop plants. Although the past decade has seen great progress in exploiting seed priming for crop protection, we still have a long way to go towards fully understanding the mechanism of plant immune memory as well as its application in sustainable agriculture. For instance, most of our knowledge about the molecular mechanism of plant defense priming comes from the study of model plants like *Arabidopsis*, establishment and maintenance mechanisms of plant defense priming in crop plants is poorly understood. Furthermore, seed priming has been widely reported on crop protection against pathogenic microbes, but its effectiveness against herbivores is less documented. In addition, degradation of thermomemory-associated heat shock proteins (HSPs) by autophagy contributes to erasing thermomemory in *Arabidopsis*, but the resetting mechanism of plant immune memory remains to be disclosed (Hilker and Schmülling, 2019; Sedaghatmehr et al., 2019). With the advance in the knowledge of plant immune memory and the development of priming methodology, exploiting seed priming would provide new avenues for better crop protection in future agriculture.

## Author contributions

CC, ZY, and PZ wrote this manuscript. All authors have read and agreed to the published version of the manuscript.

## Funding

This work was funded by the National Natural Science Foundation of China (31701412), the Natural Science Foundation of Shandong Province (ZR2017BC109), Qingdao Science and Technology Bureau Fund (17-1-1-50-jch), and Qingdao University Fund (DC1900005385).

## Conflict of interest

The authors declare that the research was conducted in the absence of any commercial or financial relationships that could be construed as a potential conflict of interest.

## Publisher’s note

All claims expressed in this article are solely those of the authors and do not necessarily represent those of their affiliated organizations, or those of the publisher, the editors and the reviewers. Any product that may be evaluated in this article, or claim that may be made by its manufacturer, is not guaranteed or endorsed by the publisher.
